# Quantitative ultrasound radiomics for therapy response monitoring in patients with locally advanced breast cancer: Multi-institutional study results

**DOI:** 10.1371/journal.pone.0236182

**Published:** 2020-07-27

**Authors:** Karina Quiaoit, Daniel DiCenzo, Kashuf Fatima, Divya Bhardwaj, Lakshmanan Sannachi, Mehrdad Gangeh, Ali Sadeghi-Naini, Archya Dasgupta, Michael C. Kolios, Maureen Trudeau, Sonal Gandhi, Andrea Eisen, Frances Wright, Nicole Look-Hong, Arjun Sahgal, Greg Stanisz, Christine Brezden, Robert Dinniwell, William T. Tran, Wei Yang, Belinda Curpen, Gregory J. Czarnota

**Affiliations:** 1 Department of Radiation Oncology, Sunnybrook Health Sciences Centre, Toronto, Canada; 2 Department of Radiation Oncology, University of Toronto, Toronto, Canada; 3 Physical Sciences, Sunnybrook Research Institute, Toronto, Canada; 4 Department of Medical Biophysics, University of Toronto, Toronto, Canada; 5 Department of Electrical Engineering and Computer Sciences, Lassonde School of Engineering, York University, Toronto, Canada; 6 Department of Physics, Ryerson University, Toronto, Canada; 7 Medical Oncology, Department of Medicine, Sunnybrook Health Sciences Centre, Toronto, Canada; 8 Department of Medicine, University of Toronto, Toronto, Canada; 9 Surgical Oncology, Department of Surgery, Sunnybrook Health Sciences Centre, Toronto, Canada; 10 Department of Surgery, University of Toronto, Toronto, Canada; 11 Department of Medical Oncology, Saint Michael's Hospital, University of Toronto, Toronto, Canada; 12 Department of Radiation Oncology, Princess Margaret Hospital, University Health Network, Toronto, Canada; 13 Department of Radiation Oncology, London Health Sciences Centre, London, Canada; 14 Department of Oncology, Schulich School of Medicine and Dentistry, Western University, London, Ontario, Canada; 15 Evaluative Clinical Sciences, Sunnybrook Research Institute, Toronto, Canada; 16 Department of Diagnostic Radiology, University of Texas, M.D. Anderson Cancer Center, Houston, Texas, United States of America; 17 Department of Medical Imaging, Sunnybrook Health Sciences Centre, Toronto, Canada; 18 Department of Medical Imaging, University of Toronto, Toronto, Canada; Fondazione IRCCS Istituto Nazionale dei Tumori, ITALY

## Abstract

**Background:**

Neoadjuvant chemotherapy (NAC) is the standard of care for patients with locally advanced breast cancer (LABC). The study was conducted to investigate the utility of quantitative ultrasound (QUS) carried out during NAC to predict the final tumour response in a multi-institutional setting.

**Methods:**

Fifty-nine patients with LABC were enrolled from three institutions in North America (Sunnybrook Health Sciences Centre (Toronto, Canada), MD Anderson Cancer Centre (Texas, USA), and Princess Margaret Cancer Centre (Toronto, Canada)). QUS data were collected before starting NAC and subsequently at weeks 1 and 4 during chemotherapy. Spectral tumour parametric maps were generated, and textural features determined using grey-level co-occurrence matrices. Patients were divided into two groups based on their pathological outcomes following surgery: responders and non-responders. Machine learning algorithms using Fisher’s linear discriminant (FLD), *K*-nearest neighbour (*K-*NN), and support vector machine (SVM-RBF) were used to generate response classification models.

**Results:**

Thirty-six patients were classified as responders and twenty-three as non-responders. Among all the models, SVM-RBF had the highest accuracy of 81% at both weeks 1 and week 4 with area under curve (AUC) values of 0.87 each. The inclusion of week 1 and 4 features led to an improvement of the classifier models, with the accuracy and AUC from baseline features only being 76% and 0.68, respectively.

**Conclusion:**

QUS data obtained during NAC reflect the ongoing treatment-related changes during chemotherapy and can lead to better classifier performances in predicting the ultimate pathologic response to treatment compared to baseline features alone.

## Introduction

Neoadjuvant chemotherapy (NAC) is the recommended first line of treatment for locally advanced breast cancer (LABC), which encompasses relatively large primary tumours (>5 cm), or disease extension to the chest wall or skin, or extensive regional lymph node metastases. The purpose of NAC is to downstage the tumour, which can increase rates of breast-conserving surgery. Also, it can facilitate the prediction of the biological behaviour of the disease with better survival observed for patients having a good response [1]. LABC is associated with a relatively poor prognosis having a median overall survival of 4.8 years [2]. Pathological complete response (pCR) with NAC is associated with improved disease-free survival and better overall survival in specific molecular groups [3–5]. In a study of 1,730 patients, those who achieved pCR had an estimated 10-year survival of 91% compared to 45% for non-responders [5]. However, only about 22% of patients who undergo NAC attained pCR, with the initial disease stage serving as a predictor of outcome [4, 6]. Although NAC is the standard first course of treatment for LABC, there are still uncertainties with regards to the optimal treatment regimen for individual patients [3].

It is crucial to develop a method to predict or identify NAC response early during treatment to assess potential treatment effectiveness to ensure a favourable outcome. Typically, treatment response is evident after several weeks to months after the initiation of therapy and is generally evaluated through clinical examination and the use of various imaging modalities like computed tomography (CT), magnetic resonance imaging (MRI), or positron emission tomography (PET). Quantitative ultrasound (QUS) imaging is non-invasive, inexpensive, and portable with excellent patient compliance, and its use in increasingly utilized in medicine. It exploits endogenous tissue elastic properties at a cellular level, using information that is otherwise lost in a standard ultrasound B-mode image [7]. Conventional B-mode ultrasound primarily relies on morphological features in tissue characterization. However, it is unable to capture a large amount of information about the internal tissue structure. Correlation of response to treatment with ultrasound b-mode images alone compared to ground truth has not shown good agreement[8]. QUS utilizes raw radiofrequency (RF) signal produced from ultrasound backscatter, which is sensitive to tissue microstructure, including cell nuclei [9–12]. These features make QUS a promising non-invasive imaging tool in medicine, aiding in diagnosis and treatment response monitoring. Oelze *et al*. have used QUS in soft tissue analysis and to characterize benign from malignant tumours in rodent models of breast cancer[11, 13, 14]. They have also shown that QUS can be used to detect breast cancer micrometastasis in excised lymph nodes[15]. Kilmonda *et al*. have also found that QUS features can be used to characterize breast imaging, reporting and data system scores in breast tissue [16]. Measurements of the backscatter coefficient (BSC) and other parameters have been used to diagnose liver disease with 87% sensitivity in a cohort of 204 patients [17]. Recently, in another application, a combination of QUS parameters showed promise in detecting prostate cancer with the hope of developing a diagnostic-guidance and treatment tool [18]. Similar work has also been conducted using data from the breast tissue of 78 patients. Lesions were classified as benign or malignant based on QUS data with a resulting sensitivity and specificity of 96% and 84%, respectively [19]. Studies have also demonstrated that changes in QUS parameters reflect cell death [10, 20–22]. Similarly, QUS parameters and textural analyses have been described to predict and monitor response to NAC in patients with LABC [23–27]. The current study was undertaken to explore the effectiveness of the QUS-radiomics obtained during NAC in predicting treatment response from a multi-institutional cohort.

The clinical goal of the study is to develop a radiomics model to predict response early into the course of treatment. The development of such non-invasive imaging biomarkers can pave the way towards precision oncology. Based on the results from a previous single institutional study, we are currently undertaking a radiomics-based adaptive chemotherapy randomized trial, where there is a provision of switching ineffective chemotherapy regimens based on QUS-models (clinicaltrials.gov study identifier NCT04050228). The presented study was extended to multiple institutions to study the applicability of such radiomics model in a diverse group of patients from different institutions.

## Materials and methods

### Patient selection and treatment

The study involved the following institutions: Sunnybrook Health Sciences Centre (Toronto, Canada), MD Anderson Cancer Centre (Texas, USA), and Princess Margaret Cancer Centre (Toronto, Canada). The study protocol was reviewed and approved by the Sunnybrook Health Sciences Centre research ethics board, the MD Anderson institutional review board and the University Health Network research ethics board. Patients were accrued after obtaining informed written consent. The study had been registered with the clinicaltrials.gov registry (NCT04134780).

Recruitment first involved the screening of potential patients to confirm eligibility. Patients with a biopsy-confirmed diagnosis of primary breast cancer without distant metastasis and decided to be treated with upfront neoadjuvant chemotherapy were considered eligible for the study. Patients were also ensured to have a life expectancy of at least 6 months, a primary tumour with a measurable size on the US and an ECOG score of 0 or 1. Exclusion criteria for this study included skin involvement, in-situ breast implants, allergies to chemotherapy agents or similar compounds and any severe medical or psychiatric comorbidities. Eligible patients were approached and given a detailed description of the study. Those interested were given a consent form to review and the opportunity to ask questions. Patients who were willing to participate had signed an informed consent form. Recruitment for this study was conducted between June 2015 and June 2018. Evaluation for human epidermal growth factor receptor (HER2), estrogen receptor (ER), and progesterone receptor (PR) status was done according to standard practice. The treating oncologist determined the specific chemotherapy regimen for each patient. However, NAC for most of the patients consisted primarily of anthracycline and taxane-based drugs. Following the completion of NAC, either breast-conserving surgery or mastectomy was decided by the patient and the treating surgeon/ oncologist. Adjuvant treatment with radiation, targeted therapy, endocrine therapy was administered as per standard institutional guidelines.

### Response criteria

Pathologic response was determined after patients had completed NAC and had undergone a lumpectomy or mastectomy. Pathologic review regarding the treatment response was conducted by the pathologist with expertise in breast pathology from individual institutions. The surgical specimen was assessed for residual disease, including cellularity and the presence of ductal carcinoma *in situ* (DCIS). Other clinical information was retrieved from the participant’s electronic medical record. For this study, a modified response criterion was applied [24]. Outcomes were classified into a dichotomous criterion of the responder (R) or non-responders (NR). Patients were considered responders if they had a complete pathological response (pCR), noted cellularity of “very low” by the pathologist, or a decrease in tumour size by greater than 30%. Patients with progressive disease or a tumour size decrease of less than 30% were classified as non-responders.

### Instrumentation and data acquisition

Participants were scanned before receiving their first dose of NAC (pretreatment) and after week 1 and week 4 of their treatment. Ultrasound scans were performed, targeting the primary breast tumour while the patient was in a supine position. Among all the patients, 42 were scanned using a Sonix RP clinical system (Ultrasonix, Vancouver, Canada), and 17 were studied using a GE LOGIC E9 system (GE Healthcare, Milwaukee, Wisconsin, USA). Scans acquired using the Sonix RP clinical system utilized an L14-5/60, linear array transducer with a center frequency of 6.3 MHz, and a bandwidth range of 3.0–8.5 MHz. RF data was obtained at a sampling rate of 40 MHz to produce an image with depth and width of 4 cm and 6 cm, respectively.

Similarly, scans using the GE LOGIC E9 system utilized an ML6-15 matrix linear array transducer probe with a center frequency of 7 MHz and a bandwidth range of 4.5–9.9 MHz. RF data were acquired at a sampling frequency of 50 MHz to produce an image of 4 cm by 5.5 cm depth and width, respectively. B-mode images were acquired simultaneously for both systems. QUS Data equivalence between both systems has recently been demonstrated [28].

### QUS data processing

QUS parameters were calculated from a selected region of interest (ROI) corresponding to the primary tumour. The ROI was manually generated around the identified tumour from the RF image planes using B-mode images. QUS spectral parameters were acquired from the normalized, frequency-dependent power spectrum of the RF data. A sliding window analysis was performed on a pixel by pixel basis within the ROI with a window size of 2 x 2 mm^2^ to include approximately 10 ultrasound wavelengths and overlap in both the lateral and axial direction of 92% [23, 26, 29, 30].

A Fourier transform was applied to create a frequency-dependent power spectrum, which was then normalized to a tissue-mimicking phantom. Normalization of the power spectrum controls for system transfer effects, diffraction artifacts, transducer beam formation, and depth-related attenuation to analyze QUS data in a system-independent manner [31, 32]. The phantom was composed of a homogeneous medium of agar-embedded glass beads and had breast tissue-like acoustic properties. Phantom data were obtained from each ultrasound system with the same settings used during patient data acquisition.

Linear regression analysis was applied to the normalized power spectrum over the -6 dB bandwidth [9, 33]. Spectral attenuation correction of the power spectrum was carried out by applying an attenuation coefficient estimate (ACE) [34]. The ACE was calculated using a reference phantom method wherein the rate of change of the spectral magnitude through the sample was estimated relative to the measured attenuation coefficient of reference medium [27, 35]. From the attenuation compensated normalization spectrum, spectral parametric maps were generated for each selected ROI, including the mid-band fit (MBF), 0-MHz spectral intercept (SI), and spectral slope (SS) from the line of best fit of the frequency-dependent power spectrum. Both MBF and SI reflect the shape, size, quantity, organization, and elastic properties of ultrasound scatterers, whereas SS is predominantly affected by scatterer shape and size [26, 33]. Mean QUS parameters were calculated by averaging the values within the maps.

Additionally, ultrasound backscatter parameters were estimated from the theoretical backscatter coefficient (BSC) [26, 36]. BSC parametric maps were calculated from the normalized power spectrum and fit a spherical Gaussian model. The Gaussian model describes the cell as the entire scatterer with the highest acoustic impedance in the center [37, 38]. The measured backscatter coefficient from the selected ROI was fitted to a theoretical BSC using a least-squares method. Subsequently, the average scatterer diameter (ASD) and average acoustic concentration (AAC) were derived [38]. The AAC is related to the density and elastic properties of the scatterer [26]. Mean spacing among scatterers (SAS) of the data was quantified from an estimated power spectrum derived by applying an autoregressive model. The SAS was calculated from the modelled estimate based on Burg’s recursive algorithm [32, 39, 40].

To reduce the dimensionality of data, and to extract discriminative features, textural features from each of the parametric maps were acquired using grey-level co-occurrence matrices (GLCM) [41, 42]. This measure is based on the spatial relationship of neighbouring pixels. Second-order statistics were subsequently computed on derived GLCMs, including determining contrast (CON), correlation (COR), homogeneity (HOM), and energy (ENE). The parameter CON is the difference of intensity levels for a set of pixels, COR is the intensity correlation between pixel pairs, HOM measures the incidence of pixel pairs for intensity, and ENE measures the power of the frequency for an occurrence of pixel pairs and reflects image textural uniformity. A total of 31 parameters were obtained from the QUS data from each experimental assessment time and considered for submission to multi-parametric classifier models, in addition to values or parameters from the pretreatment QUS data.

### Data analysis and classification

Statistical analysis was conducted to compare the QUS parameters between responding and non-responding patients. The Shapiro-Wilk test was used to ensure distribution normality. Normally distributed QUS data were analyzed using a one-way analysis of variance (ANOVA), whereas the Mann-Whitney U test was used for data that were not normally distributed. A p-value of <0.05 was considered significant. Machine learning classification analyses of the QUS data and texture were performed using MATLAB R2011b (The Math Works Inc., USA). Data balancing was done to prevent biasing classifiers with data samples from the majority class. Random under-sampling was used for the majority class to make it equal in size to the minority class. Fisher’s linear discriminant (FLD), *K*-nearest neighbour (*K-*NN), and support vector machine-radial based function (SVM-RBF) classifiers were used to develop a model to predict the pathological response from the 62 parameter values for each experimental assessment time (combining baseline data with week 1 and 4). FLD and SVM-RBF Analyses were conducted using a balanced dataset where the response groups had an equal number of samples, whereas *K-*NN analysis was performed on unbalanced data. Feature selection amongst the 62 parameters was conducted to ameliorate the curse of dimensionality that occurs from a limited sample set [43]. To elaborate further, as a rule of thumb, 10 samples per class are needed for each feature added to the classification model to avoid a peaking phenomenon [44, 45]. Sequential forward selection (SFS), therefore, was used in a wrapper framework to address this problem [44]. Each prediction model was tested through leave-one-out (LOO) cross-validation at the subject level wherein the test-sample was excluded from the training set and from tuning the classifier parameters and feature selection. A minimum of one feature and a maximum of 4 features were selected to generate a multi-parametric prediction model. Multiple iterations using different random under-sampled majority classes were performed, and the results were averaged and reported. The performance of each prediction model was evaluated by measuring accuracy, sensitivity, specificity, and area-under-the-curve (AUC) of the receiver-operating characteristic (ROC) curve.

## Results

### Patient characteristics and treatment outcomes

A total of 59 patients were available for the current study analysis. Relevant patient-specific information is listed in Table 1. The age of the patients ranged from 27 to 74 years, with a median of 52 years. Among all the patients, 56 were administered anthracycline and taxane-based chemotherapy; 40 were given doxorubicin, cyclophosphamide, and paclitaxel (AC-T), and 16 had fluorouracil, epirubicin, cyclophosphamide and docetaxel (FEC-D). One patient received Taxol without an anthracycline. One patient received cisplatin, and another was administered Taxol and cyclophosphamide. The molecular status of the patients was determined from the initial biopsy: 41 patients were ER-positive, 33 were PR positive, and 19 were HER2 positive. All the HER2 positive patients received trastuzumab, and 3 of them also received pertuzumab as per institutional treatment practice. Patients had undergone surgery 3.1 to 6.6 months (mean 4.8 ± 0.7 months) after beginning NAC. The time between patient's last chemotherapy and surgery was 0.8 to 2.7 months (mean 1.3 ± 0.5 months). Of the 59 study participants, 35 were classified as responders, while 24 were non-responders. Pre-treatment median tumour size in non-responders was 3.75 cm (1.80 cm to 8.80 cm) and 3.50 cm (1.80 cm to 15.70 cm) post-treatment. Whereas, pre-treatment median tumour size in responders were 3.70 cm (1.20 cm to 11.60 cm) and 1.55 cm (0.00 cm to 10.0 cm) post-treatment. Patient characteristics on an individual patient basis are presented in S1 Table.

**Table 1:** Relevant patient-specific information and disease characteristics.

Patient Characteristics (n = 59)	Frequency
**Age**	
Median	52
Range	27–74
**Sex**	
Female	58
Male	1
**Initial tumour size**	Median 3.70 cm
	Range (1.2–11.6) cm
**Molecular Markers**	
ER+	69%
PR+	56%
HER2+	32%
TNBC	20%
**Histological Type**	
IDC	80%
ILC	12%
IMC/Other	8%
**Chemotherapy**	
AC-T	68%
FEC-D	27%
Taxol, no anthracycline	1.7%
Trastuzumab	32%
Pertuzumab	5%
Cisplatin	1.7%
Carboplatin, Taxol	1.7%
**Treatment Response**	
Responder	59%
Non- Responder	41%

**Abbreviations:** ER+/PR+: Estrogen/Progesterone-receptor status, HER2+: Human epidermal growth factor receptor-2 status, TNBC: Triple-negative breast cancer, IDC: Invasive ductal carcinoma, ILC: Invasive lobular carcinoma, IMC: Invasive mammary carcinoma, AC-T: Doxorubicin (Adriamycin) and Cyclophosphamide followed by Taxol, FEC-D: 5-Fluorouracil, Epirubicin, Cyclophosphamide, and Docetaxel, Trastuzumab (Herceptin): Monoclonal antibody.

### QUS analysis

**Fig 1:**
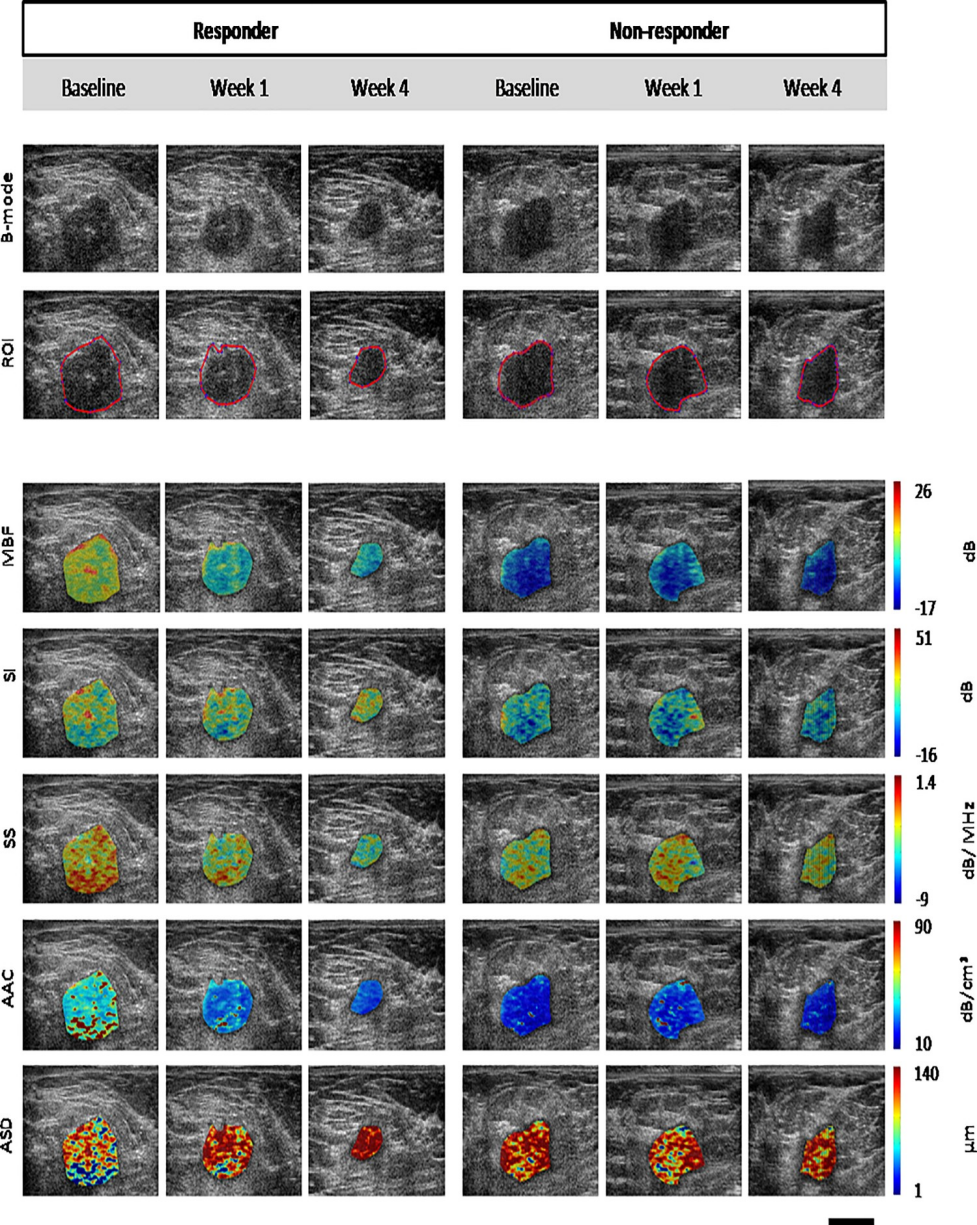
Ultrasound B-mode and QUS-derived parametric maps for representative responder and non-responder patients (responder—left, non-responders—right panel) acquired at baseline, and weeks 1 and 4 of treatment. **Abbreviations**: MBF (dB): mid-band fit, SS (dB/MHz): spectral slope, ASD (μm): average scatterer diameter, AAC (dB/cm^3^): average acoustic concentration, SI (dB): spectral intercept. Scale bar represents 2 cm.

Fig 1 presents representative ultrasound B-modes images, and corresponding ROIs and QUS parametric maps between a responder and non-responder at baseline (pretreatment), week 1, and week 4.

Tumours presented as typical hypoechoic areas that were well defined. Non-responders demonstrated no significant size changes, whereas, for some responding patients, there were evident tumour size changes in particular at 4 weeks. In addition, in patients responding to treatment, there were obvious changes in QUS data as early as week one. This was more obvious in specific parametric maps compared to other parametric maps. Parametric maps also demonstrated changing heterogeneity, particularly in responding patients (see Fig 1). in contrast, parametric maps in non-responding patients appeared mostly without change.

**Fig 2:**
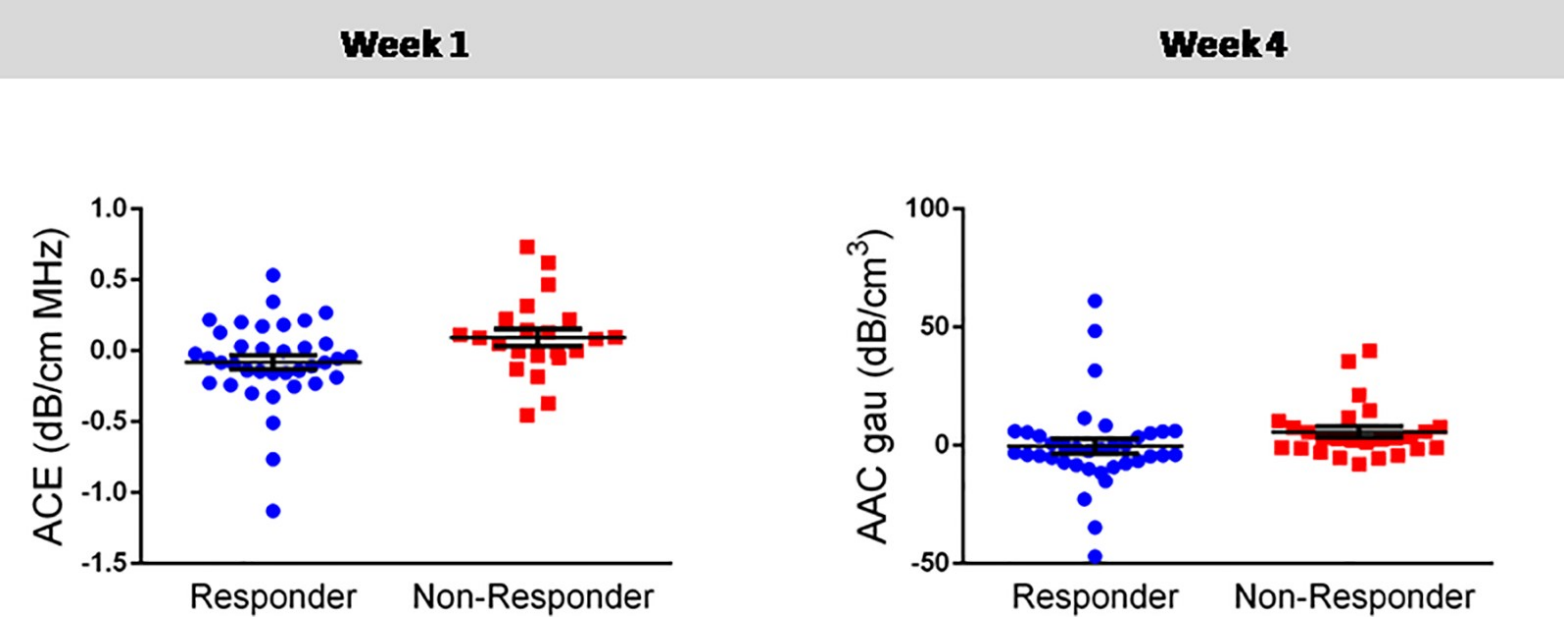
Statistically significant QUS parameters between responders and non-responders at weeks 1 and 4 of neoadjuvant chemotherapy. Error bars represent ± one standard error of the mean, and significance was determined at *p* < 0.05. **Abbreviations**: AAC (dB/cm^3^): average acoustic concentration, ACE: Attenuation Coefficient Estimate.

**Fig 3:**
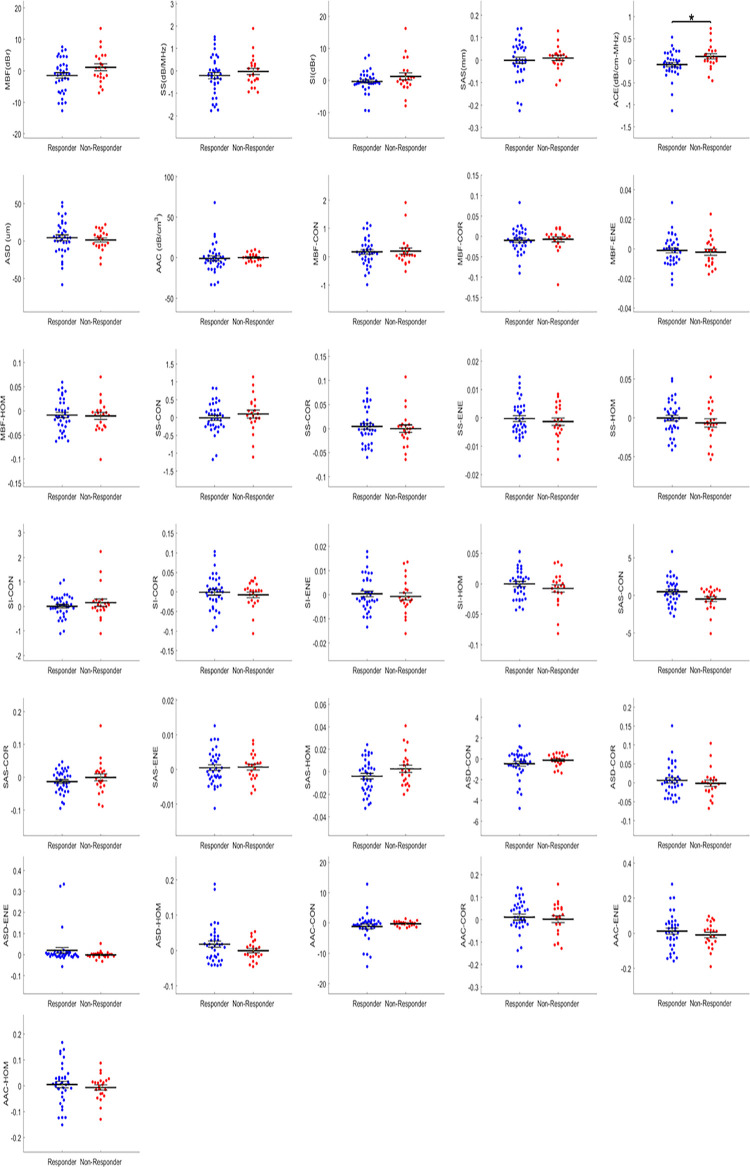
Scatter plots of QUS parameters comparing responders and non-responders at week 1. Error bars represent ± one standard error of the mean, and significance was determined at *p* < 0.05.

**Fig 4:**
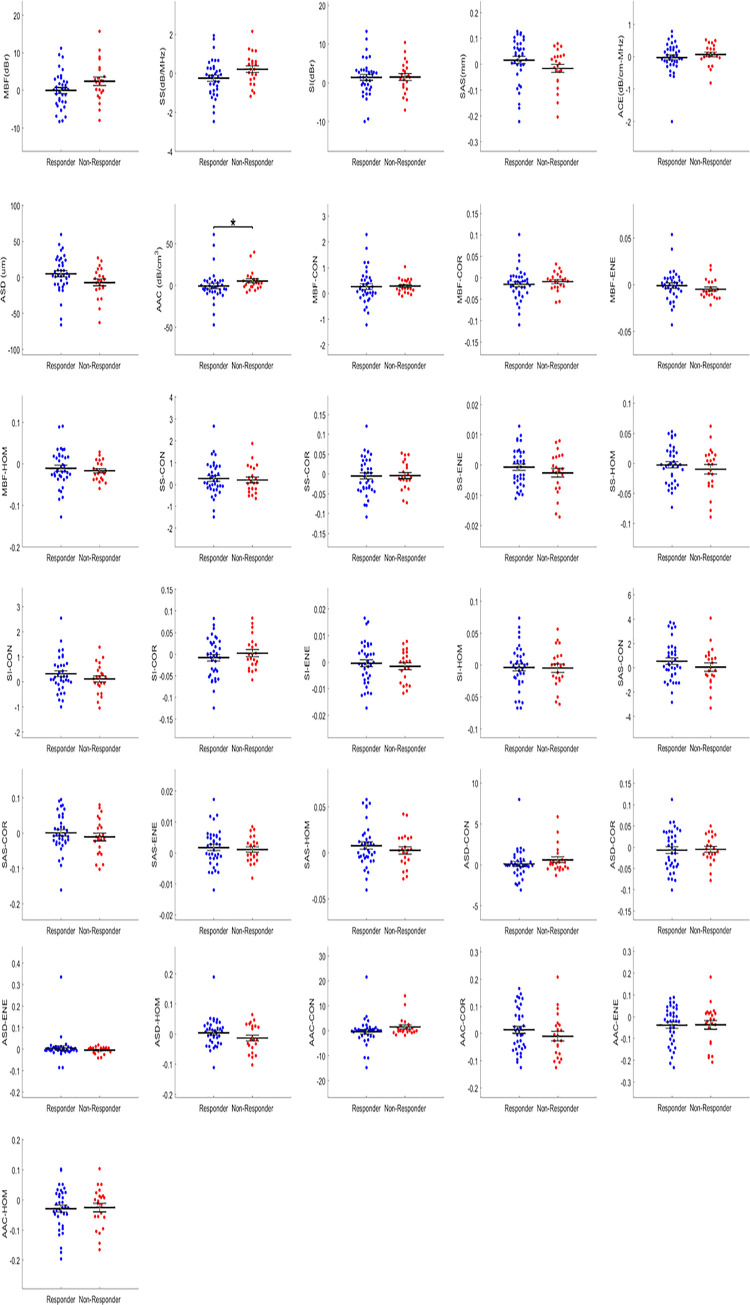
Scatter plots of QUS parameters comparing responders and non-responders at week 4. Error bars represent ± one standard error of the mean, and significance was determined at *p* < 0.05.

Statistical analysis identified two mean-value parameters that were significantly different on their own between responders and non-responders. These were the ΔACE (*p* = 0.018) at week 1 and ΔAAC (*p* = 0.023) at week 4. At week 1, the mean value of ΔACE between responders and non-responders were -3.00 dB/cm-MHz and -3.70 dB/cm-MHz, respectively. At week 4, for ΔAAC, the mean value was 5.52 dB/cm^3^ and 2.58 dB/cm^3^ for the responders and non-responder groups, respectively. Table 2 shows statistical analysis results with mean values of the significant parameters, standard errors, and corresponding *p*-values. Fig 2 displays statistically significant parameters in a scatter plot with each point representing every responder and non-responder patient, the extended midline identifies the mean, and error bars represent the standard error of the mean. Figs 3 and 4 demonstrate the distribution of data as scatter plots comparing responders and non-responders for all 31 QUS parameters and texture-related parameters at weeks 1 and 4, respectively. As is evident, the majority of parameters at both weeks 1 and 4 were not statistically significant on their own. Changes were evident between parameters at week1 and week 4.

**Table 2:** Statistically significant QUS mean values and textural parameters between response groups at week 1 and week 4 into neoadjuvant chemotherapy.

Parameter	Mean ± SEM (R)	Mean ± SEM (NR)	*p*-value
**Week 1**			
ΔACE (dB/cm-MHz)	-3.00 ± 0.18	-3.70 ± 0.20	0.018
**Week 4**			
ΔAAC (dB/cm^3^)	5.52 ± 0.93	2.58 ± 0.54	0.023

**Abbreviations:** SEM: standard error of the mean, ACE: Attenuation Coefficient Estimate, AAC: Average Acoustic Concentration.

### Classifier performances

Fisher's linear discriminant analysis (FLD), *K*-nearest neighbour (*K*-NN), and support vector machine (SVM) learning algorithms used QUS parameters to generate models that could classify patient treatment response. Table 3 identifies optimal classification models from each algorithm that were generated using multiple features from baseline (pretreatment), week 1, and week 4 QUS data. Four of the best classifying features were used in model construction. For all the models, the classifier performance was improved when baseline QUS features were combined with week 1 and week 4, respectively. The performance was lower when baseline, week1 or week 4 features were used alone. Among all classification models, the SVM-RBF classification had the highest response prediction accuracy, AUC, and F1-score values at 81%, 0.87, and 0.81 at both week 1 and week 4. At week 1, the selected best features were AAC_W0_, ΔAAC-CON, and ASD-CON_W0_. The sensitivity and specificity for this model were 83% and 79%, respectively. At week 4, the selected best features were AAC_W0_, ΔSI, AAC-CON_W0_, and AAC-CON_W0_. The sensitivity and specificity for the week 4-SVM-RBF model were 80% and 82%, respectively.

**Table 3:** Optimal multivariate-feature classification analysis using machine learning algorithms in week 1 and week 4 during neoadjuvant chemotherapy.

Classifier	%S_n_	%S_p_	%Acc	AUC	F1-score	Features
**Baseline**						
FLD	56	71	61	0.60	0.63	SI-COR_W0_
*K*-NN	83	53	75	0.68	0.65	SI-ENE_W0_
						MBF-ENE_W0_
						ASD-ENE_W0_
SVM-RBF	81	65	76	0.68	0.72	MBF_W0_
						AAC-CON_W0_
						SS_W0_
						MBF-CON_W0_
**Week 1**						
FLD	82	63	70	0.73	0.71	ΔACE
						AAC_W0_
						SS-CON_W0_
*K-*NN	82	65	71	0.71	0.72	AAC_W0_
						ΔSAS
						ΔMBF-COR
SVM-RBF	83	79	81	0.87	0.81	AAC_W0_
						ASD-CON_W0_
						ΔAAC-CON
**Week 4**						
FLD	74	69	71	0.71	0.72	AAC_W0_
						SAS-COR_W0_
						ΔSAS
						ASD-ENE_W0_
*K-*NN	77	70	73	0.74	0.74	SAS_W0_
						ΔSI-HOM
						ΔMBF-HOM
SVM-RBF	80	82	81	0.87	0.81	AAC_W0_
						ASD-CON_W0_
						ΔSI
						AAC-CON_W0_

**Abbreviations**: S_n_: sensitivity; S_p_: specificity, AUC: area under curve, Acc: accuracy, FLD: Fisher’s linear discriminant, *K-*NN: *K-*nearest neighbours, SVM-RBF: support vector machine with radial basis function kernel, AAC (dB/cm^3^): average acoustic concentration, SS (dB/MHz): spectral slope, SI (dBr): spectral intercept, SS (dB/MHz): spectral slope, SAS (mm): spacing among scatterers, ASD (μm): average scatterer diameter, MBF (dB): mid-band fit, HOM: homogeneity, ENE: energy, CON: concentration

**Fig 5:**
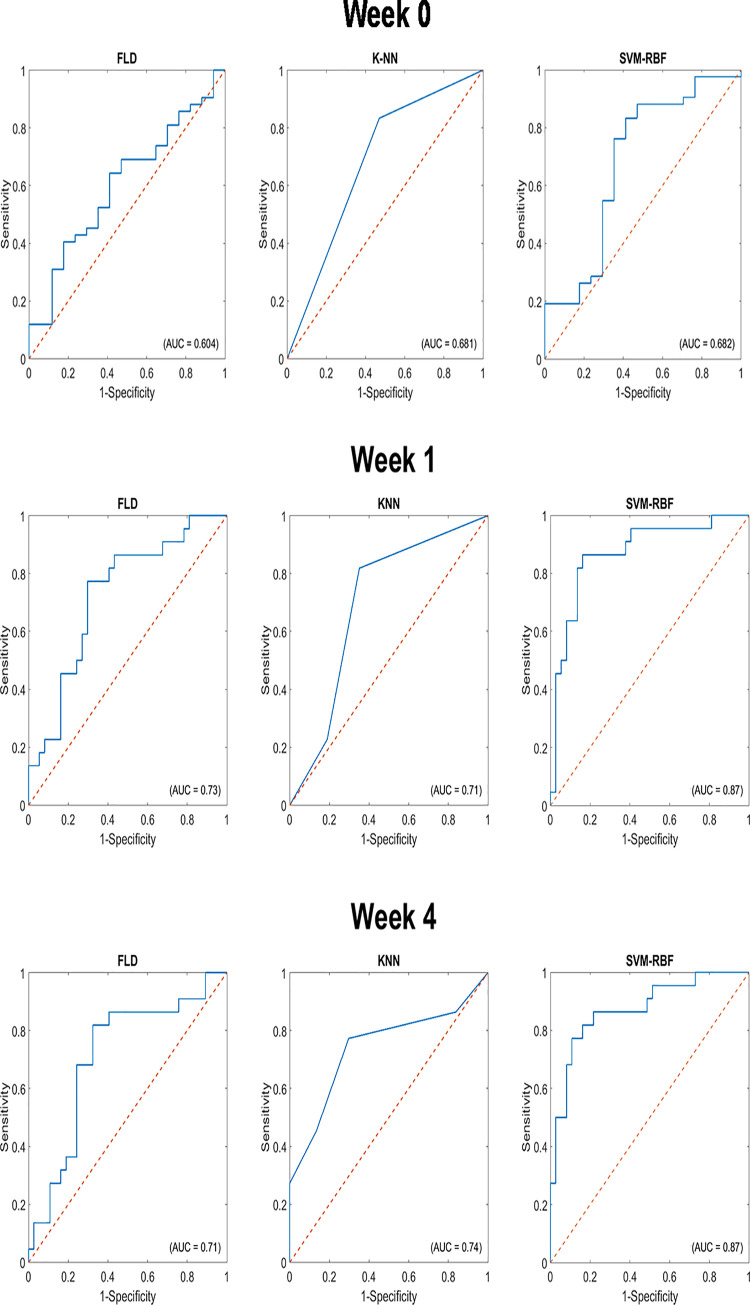
Receiver operating characteristic curves of QUS feature selection using machine learning algorithms from data acquired before initiation and at weeks 1 and 4 of neoadjuvant chemotherapy. Area under curve (AUC) values are indicated in the respective curves.

Following the SVM-RBF response models, *K*-NN at week 4 had an accuracy of 73%, AUC of 0.74, and F1-Score of 0.74. This model performed best using three features: SAS_W0_, ΔSI-HOM, and ΔMBF-HOM. Fig 5 shows the ROC curves for the classification models generated at week 1 and week 4, with the corresponding AUC curves identified within the curve. We had performed analysis combing week 1 and week 4 features, which did not result in any better classifier results.

## Discussion

This study aimed to investigate the use of QUS and texture analysis to monitor the LABC tumour response to NAC using a patient cohort recruited from three different healthcare institutions. Previous work involved only single institutional data and data from one scanner type, whereas the work here is a multi-institution validation of the method with different scanner types. The study presented here exploits the difference in tissue microstructure between the two groups with different biological outcomes (response to chemotherapy) using quantitative imaging and radiomic analysis. The quantitative spectral features serve as first-order imaging features that are influenced by factors like scatterer size, shape, organization of the tissue at the cellular level. Using a computational radiomic approach through texture analysis (second-order features) reveals further information related to intratumoral heterogeneity, which is known to be an important determinant of treatment response and clinical outcomes. In a previous study, we had shown the addition of texture values compared to QUS spectral parameters alone resulted in better performances of the classifier models [23]. In this study, based on the changes in QUS and texture features 1 week and 4 weeks after the start of treatment, multi-feature discriminant algorithms were developed using machine learning classifiers to predict patients having a response to NAC. We have demonstrated the inclusion of QUS features before treatment significantly improves the accuracy of the response-monitoring models. In addition, the combination of week 1 and week 4 features together resulted in better performance at either time at predicting clinical response 4–5 months later at the end of patient NAC.

All algorithms derived from the FLD, *K*-NN, and SVM-RBF classifiers achieved good differentiation accuracy greater than 70%. The SVM-RBF algorithm demonstrated the highest accuracy of 81% at both week 1 and week 4. For all three algorithms, the accuracy was superior or relatively the same at week 4 compared to week 1. The accuracy of the FLD algorithm increased from 70% to 71% and *K*-NN algorithm institutions from 71% to 73% at week 1 and week 4, respectively. This highlights the importance of continuous therapy monitoring, as there may be a time-dependent improvement of accuracy since data is derived from tumours, which may be faster responding than others. This can be potentially attributed to the ongoing changes in the microenvironment in response to treatment from cell death, reflected as changes in ultrasound backscatter parameters. For the best performing algorithm (SVM-RBF), the selected features were AAC_W0_, ASD-CON_W0_, and ΔAAC-CON at week 1, and AAC_W0_, ASD-CON_W0_, ΔSI, and AAC-CON_W0_ at week 4.

In addition to multi-parametric monitoring algorithms, the statistical difference of the QUS and texture features with the response to NAC was analyzed. As evident in Fig 2, the ΔACE (*p* = 0.018) and ΔAAC (*p* = 0.023) parameters were determined to have a statistically significant difference between responders and non-responders at week 1 and week 4, respectively. These significant QUS parameters reflect the biological changes within the tumour as the ACE accounts for tissue composition and stiffness, and the AAC reflects the microstructural change related to the concentration of acoustics scatterers as a result of cell death [45]. Furthermore, the significance of the ACE feature in week 1 is consistent with the observations made in previous clinical studies [30, 46].

The significance of the work here is the demonstration of the use of QUS parameters across institutions and devices. Equivalency between the RF data analysis across these devices has previously demonstrated. Other imaging modalities, including MRI and PET, have also been shown to be useful for response monitoring. Due to the sensitivity of these methods to tissue microvasculature, basic pharmacokinetic parameters and texture features from dynamic contrast enhanced-MRI (DCE-MRI) have been studied as early markers of pathological response [47–50]. Likewise, the apparent diffusion coefficient obtained from diffusion-weighted imaging-MRI (DWI-MRI) has been found to correlate well with NAC response [51–53]. The role of functional imaging, including positron emission tomography (PET), has been investigated in predicting response to NAC. A recent study using PET/computed tomography (CT) showed a difference in the standardized uptake value of ^18^F-FDG between pCR and non-pCR at baseline [54]. Another emerging method is diffuse optical spectroscopy (DOS) [55–57], which can detect tissue hemoglobin and lipid concentration. Baseline parameters before initiation of treatment have been used able to predict pathological response to NAC with an accuracy of 89% in a cohort of 37 LABC patients [56]. Overall, these studies support the importance of the early detection of functional, microstructural differences over macroscopic size changes as a predictor or early marker of treatment response. Molecular events related to tumour cell death from therapy occur in the early stages of treatment, before later stage changes in tumour size [27, 58–60]. Nevertheless, DCE-MRI and PET require the administration of exogenous agents, and PET/CT uses ionizing radiation, which limits repetitive imaging [61, 62]. Standard imaging modalities use the morphological changes associated with the treatments to evaluate response, which is not manifested until a few weeks or months of treatment. Contrarily, quantitative imaging analysis can provide insights into the structural changes of the tumour much ahead of the morphological changes. As it is known with the initiation of anticancer therapies, cell death starts in the first few hours and the final structural change is from the continuous ongoing process with an accumulation of cell death. Symmans et al. have shown that early cell death during treatment is a good indicator of treatment outcome [63].

The early identification of responders and non-responders to NAC using the QUS-based monitoring technology demonstrated here has the potential to improve response-guided adaptive therapy. Currently, tumour response is assessed by tumour size change and histological results after surgery. Using these, it may take many months to discern if the tumour has responded to NAC. Using QUS, the response can be determined as early as 1 to 4 weeks into treatment with good accuracy. Knowledge of treatment outcome could aid in deciding if a patient should continue with treatment or switch to a more effective treatment or undergo salvage therapy. The importance of adaptive therapy has been demonstrated by von Minckwitz *et al*. [64], which has shown that patients receiving response guided chemotherapy exhibited significantly longer disease-free survival compared to patients who received conventional chemotherapy. In that study, the response was determined based on sonography obtained 6 weeks after the initiation of chemotherapy. The QUS technique here can identify responders and non-responders at an earlier time point (within 1^st^ week of treatment). Therefore, it may enable an earlier switch to adaptive therapy, which may further improve the survival outcome.

The limitation of this study is the relatively small number of participating institutions and enrolled patients. Expanding the numbers of participating institutions and patient recruitments will enable the robustness and applicability of the radiomics model, and further demonstrate the technique can be applied universally with a minimized operator and system dependency.

## Conclusion

This multi-site study demonstrates that QUS parameters and associated texture analysis can be used to develop classification algorithms that can identify responders and non-responders to NAC at early treatment times with high accuracy. This multi-site and device trial further validates the robustness of the model with a minimum operator and system dependency. The quantitative ultrasound-based response monitoring has the potential to facilitate response-guided adaptive chemotherapy at an early treatment stage to improve response in patients receiving NAC much ahead of the morphological changes detected by conventional imaging modalities or final histopathologic evaluation.

## Supporting information

S1 Table (DOCX)
